# Supplementary material for: Causal Associations Between Pre-Pregnancy Diabetes Mellitus and Pre-Eclampsia Risk: Insights from a Mendelian Randomization Study
Source: Healthcare (Basel). 2025 May 7;13(9):1085. doi: 10.3390/healthcare13091085 (PMC12072006; doi:10.3390/healthcare13091085)
Supplement: Supplementary file 1 [file healthcare-13-01085-s001.zip › sup fig legend.pdf]

Supplementary Figure S1: A forest plot presenting causal estimates from leave-one-out MR analyses of T1D on PE. The variant removed from each analysis is presented on the y-axis.Causal effect estimates are presented on the x-axis with beta (logOR) values, with error barsshowing the 95% confidence intervals (95% CI) for each estimate.

Supplementary Figure S2: A forest plot presenting causal estimates from leave-one-out MR analyses of T2D on PE. The variant removed from each analysis is presented on the y-axis.Causal effect estimates are presented on the x-axis with beta (logOR) values, with error barsshowing the 95% confidence intervals (95% CI) for each estimate.

Supplementary Figure S3: A forest plot presenting causal estimates from leave-one-out MR analyses of HbA1c on PE. The variant removed from each analysis is presented on the y-axis.Causal effect estimates are presented on the x-axis with beta (logOR) values, with error barsshowing the 95% confidence intervals (95% CI) for each estimate.

Supplementary Figure S4: A forest plot presenting causal estimates from leave-one-out MR analyses of FI on PE. The variant removed from each analysis is presented on the y-axis.Causal effect estimates are presented on the x-axis with beta (logOR) values, with error barsshowing the 95% confidence intervals (95% CI) for each estimate.

Supplementary Figure S5: A forest plot presenting causal estimates from leave-one-out MR analyses of BMI on PE. The variant removed from each analysis is presented on the y-axis.Causal effect estimates are presented on the x-axis with beta (logOR) values, with error barsshowing the 95% confidence intervals (95% CI) for each estimate.

Supplementary Figure S6: A forest plot and summary table of causal estimates from bidirectional Mendelian randomisation (MR) analyses testing for potential causal relationships between antidiabetic medications and preeclampsia (PE). Error bars show 95% CI for overall estimates (OR) from each MR method (left). IVW-RE, random-effects inverse-variance weighted.
