# Supplementary material for: Causal Associations Between Pre-Pregnancy Diabetes Mellitus and Pre-Eclampsia Risk: Insights from a Mendelian Randomization Study
Source: Healthcare (Basel). 2025 May 7;13(9):1085. doi: 10.3390/healthcare13091085 (PMC12072006; doi:10.3390/healthcare13091085)
Supplement: Supplementary file 1 [file healthcare-13-01085-s001.zip › sup table.pdf]

Supplementary Table S1. Genetic instruments used in MR analysis of T1D and preeclampsia

SNP	EA	OA	Beta	SE	p	R2	F-statistic
rs11203203	A	G	0.149971	0.0189963	2.91E-15	2.10E-03	62.32704479
rs113010081	C	T	-0.16405	0.0300091	4.59E-08	1.01E-03	29.88453674
rs12150079	A	G	0.114043	0.0200075	1.20E-08	1.09E-03	32.49014245
rs12416116	A	C	-0.164993	0.0209991	3.93E-15	2.08E-03	61.73474733
rs12927355	T	C	-0.193949	0.0199947	3.02E-22	3.16E-03	94.09039781
rs1456988	T	G	-0.111005	0.0200008	2.86E-08	1.04E-03	30.80281079
rs1574285	T	G	-0.115024	0.0190039	1.42E-09	1.23E-03	36.63460047
rs1893217	G	A	0.192024	0.024003	1.24E-15	0.002153867	64
rs229533	C	A	0.106969	0.0189945	1.79E-08	1.07E-03	31.71466584
rs2304256	A	C	-0.139032	0.0210049	3.61E-11	0.001475443	43.81151828
rs2611215	G	A	-0.167969	0.0249954	1.82E-11	0.001520731	45.15835268
rs3087243	A	G	-0.178051	0.0190054	7.36E-21	0.002951389	87.76771775
rs34593439	A	G	-0.246028	0.0330038	9.02E-14	1.87E-03	55.57010009
rs3842727	T	G	0.686966	0.0229989	4.89E-196	2.92E-02	892.1879561
rs402072	C	T	-0.142025	0.0260045	4.72E-08	0.001005012	29.82858008
rs41295121	T	C	-0.652965	0.111994	5.53E-09	0.001145165	33.99306255
rs4820830	T	C	-0.134968	0.0189955	1.20E-12	1.70E-03	50.48474486
rs4849135	G	T	0.114962	0.0209931	4.35E-08	1.01E-03	29.98855034
rs516246	T	C	0.142947	0.018993	5.22E-14	1.91E-03	56.64517909
rs56994090	C	T	-0.12897	0.0189956	1.13E-11	1.55E-03	46.09686261
rs6043409	G	A	0.126017	0.0200027	2.98E-10	1.34E-03	39.6899937
rs61839660	T	C	-0.471925	0.0359943	2.84E-39	5.76E-03	171.9010403
rs62447205	G	A	-0.116983	0.020997	2.53E-08	1.05E-03	31.0406647
rs6679677	A	C	0.635995	0.0269998	1.10E-122	1.84E-02	554.8636935
rs6691977	C	T	0.126016	0.0230029	4.30E-08	1.01E-03	30.011396
rs6827756	C	T	-0.131028	0.0190041	5.40E-12	1.60E-03	47.53720135
rs7239671	G	A	0.120978	0.0179967	1.79E-11	1.52E-03	45.18840856
rs72928038	A	G	0.179985	0.0239981	6.38E-14	1.89E-03	56.24953121
rs8056814	A	G	0.27801	0.0310012	3.03E-19	0.002704972	80.41995498
rs9585056	T	C	-0.116004	0.0210007	3.32E-08	0.001028033	30.5125418

Supplementary Table S2. Genetic instruments used in MR analysis of T2D and preeclampsia

SNP	EA	OA	Beta	SE	p	R2	F-statistic
rs10077431	A	C	-0.0487	0.0089	4.75E-08	4.57E-05	29.94180028
rs10087241	A	G	-0.0475	0.008	2.80E-09	5.38E-05	35.25390625
rs10100265	C	A	-0.0491	0.0079	6.29E-10	5.89E-05	38.62858516
rs10114341	C	T	-0.0409	0.0072	1.15E-08	4.92E-05	32.26871142
rs10401969	C	T	0.0921	0.0133	4.13E-12	7.31E-05	47.95302165
rs1050226	G	A	-0.0491	0.0074	3.34E-11	6.71E-05	44.02501826
rs1061813	A	G	-0.0429	0.0073	3.37E-09	5.27E-05	34.5357478
rs1063355	G	T	0.0709	0.0079	3.72E-19	0.00012283	80.54494472
rs10740322	A	G	0.0477	0.0085	2.11E-08	4.80E-05	31.49190311
rs10811661	C	T	-0.1569	0.0098	4.13E-58	0.000390789	256.3266347
rs10830963	G	C	0.0909	0.008	5.85E-30	0.000196871	129.1064063
rs10842994	T	C	-0.0755	0.0091	1.02E-16	0.000104975	68.83528559
rs10974438	C	A	0.0591	0.0075	3.01E-15	9.47E-05	62.0944
rs11107116	T	G	0.0467	0.0085	3.75E-08	4.60E-05	30.18532872
rs1111875	T	C	-0.0948	0.0072	3.61E-39	0.000264336	173.3611111
rs11257655	T	C	0.0737	0.0087	1.97E-17	0.000109438	71.76231999
rs1127655	T	C	-0.0438	0.0079	2.47E-08	4.69E-05	30.7393046
rs11925227	A	G	-0.0534	0.0095	2.25E-08	4.82E-05	31.59623269
rs11926707	C	T	0.0463	0.0082	1.69E-08	4.86E-05	31.88117192
rs12088739	G	A	-0.0884	0.013	9.79E-12	7.05E-05	46.24
rs12299509	G	A	0.0467	0.0073	2.09E-10	6.24E-05	40.92493901
rs12617659	T	C	-0.0685	0.0103	2.83E-11	6.75E-05	44.22895655
rs12910825	G	A	0.0517	0.0074	2.16E-12	7.44E-05	48.81099343
rs12945601	C	T	-0.048	0.008	1.72E-09	5.49E-05	36
rs12970134	A	G	0.0555	0.008	5.31E-12	7.34E-05	48.12890625
rs13234269	A	T	-0.0583	0.0078	6.98E-14	8.52E-05	55.86604208
rs13239186	T	C	0.0539	0.0085	2.70E-10	6.13E-05	40.21051903
rs13330951	G	A	-0.0456	0.0081	1.54E-08	4.83E-05	31.69272977
rs13389219	T	C	-0.0722	0.0074	2.11E-22	0.000145167	95.19430241
rs1359790	A	G	-0.0796	0.008	2.80E-23	0.000150973	99.0025
rs1496653	G	A	-0.0769	0.0088	2.57E-18	0.000116454	76.3637655
rs16988333	G	A	-0.0745	0.013	9.17E-09	5.01E-05	32.84171598
rs17086692	T	G	-0.0467	0.0084	2.48E-08	4.71E-05	30.90830499
rs17168486	T	C	0.0742	0.0094	2.18E-15	9.50E-05	62.30918968
rs17405722	A	G	0.087	0.0146	2.28E-09	5.42E-05	35.50853819
rs17411031	G	C	-0.045	0.0081	3.04E-08	4.71E-05	30.86419753
rs1758632	G	C	0.0491	0.0081	1.36E-09	5.60E-05	36.74455114
rs17631783	T	C	-0.0487	0.0089	3.95E-08	4.57E-05	29.94180028
rs17791513	G	A	-0.1027	0.0148	4.61E-12	7.34E-05	48.1523466
rs1801214	T	C	0.0903	0.0074	5.52E-34	0.000227056	148.9059533
rs1899951	T	C	-0.1118	0.0109	1.64E-24	0.000160428	105.2036024
rs2058913	T	A	-0.0491	0.0078	3.26E-10	6.04E-05	39.62541091
rs2237892	T	C	-0.096	0.0157	8.75E-10	5.70E-05	37.38894073
rs2261181	T	C	0.0985	0.0118	9.18E-17	0.000106263	69.68004884
rs2294120	G	A	-0.0443	0.0079	1.62E-08	4.80E-05	31.44512097
rs2296173	G	A	0.065	0.0087	7.66E-14	8.51E-05	55.81979125

rs2299383	T	C	0.0412	0.0073	1.49E-08	4.86E-05	31.85288047
rs243019	C	T	0.0566	0.0071	2.29E-15	9.69E-05	63.55008927
rs2493394	G	A	0.073	0.0113	1.15E-10	6.36E-05	41.73388676
rs2796441	A	G	-0.0715	0.0073	1.96E-22	0.000146292	95.93263276
rs2820426	G	A	0.0521	0.0073	1.30E-12	7.77E-05	50.93657347
rs2867125	C	T	0.0601	0.0096	4.33E-10	5.98E-05	39.19281684
rs2908282	A	G	0.0552	0.0094	4.25E-09	5.26E-05	34.48438207
rs2925979	C	T	-0.0534	0.0078	9.06E-12	7.15E-05	46.86982249
rs3217992	T	C	0.0527	0.0073	7.23E-13	7.95E-05	52.11653218
rs340874	C	T	0.0626	0.0073	8.41E-18	0.000112143	73.5364984
rs348330	A	G	-0.0487	0.0081	1.86E-09	5.51E-05	36.14830056
rs3756784	G	T	0.0505	0.0091	2.59E-08	4.70E-05	30.7964014
rs459193	G	A	0.0711	0.0083	8.81E-18	0.000111906	73.38089708
rs4622883	G	A	-0.0435	0.0078	3.02E-08	4.74E-05	31.10207101
rs4686471	C	T	0.0534	0.0081	4.28E-11	6.63E-05	43.46227709
rs4812829	A	G	0.0532	0.0095	2.44E-08	4.78E-05	31.36
rs4823182	G	A	0.0482	0.0077	3.36E-10	5.98E-05	39.18434812
rs4865796	A	G	0.053	0.0078	1.33E-11	7.04E-05	46.17028271
rs516946	C	T	0.0824	0.0085	3.16E-22	0.000143309	93.97591696
rs5215	T	C	-0.0678	0.0073	2.09E-20	0.000131545	86.26083693
rs55966194	G	C	-0.0526	0.0088	2.25E-09	5.45E-05	35.72778926
rs576674	A	G	-0.0654	0.0097	1.79E-11	6.93E-05	45.45817834
rs6059662	G	A	0.0446	0.0079	1.51E-08	4.86E-05	31.87245634
rs61953351	T	G	-0.07	0.0091	1.98E-14	9.02E-05	59.17159763
rs622217	C	T	-0.0485	0.0077	3.13E-10	6.05E-05	39.67363805
rs6494307	G	C	-0.0443	0.0078	1.67E-08	4.92E-05	32.25657462
rs6515236	C	A	-0.0504	0.0091	3.34E-08	4.68E-05	30.67455621
rs67232546	T	C	0.0596	0.0096	4.66E-10	5.88E-05	38.54340278
rs6767484	G	A	0.1209	0.0076	2.70E-56	0.000385813	253.061115
rs6785040	C	T	-0.0633	0.0111	1.26E-08	4.96E-05	32.52081812
rs6795735	T	C	-0.0558	0.0073	1.63E-14	8.91E-05	58.42822293
rs6878122	A	G	-0.0564	0.0079	1.19E-12	7.77E-05	50.96875501
rs6960043	C	T	0.064	0.0071	3.61E-19	0.00012391	81.2537195
rs7144011	T	G	0.0482	0.0085	1.64E-08	4.90E-05	32.15557093
rs7177055	A	G	0.0647	0.0079	2.75E-16	0.000102289	67.0740266
rs7240767	C	T	0.0451	0.0081	2.16E-08	4.73E-05	31.00152416
rs72802358	C	G	-0.1168	0.0133	1.97E-18	0.000117612	77.12273164
rs72892910	T	G	0.0648	0.0099	6.43E-11	6.53E-05	42.84297521
rs735949	C	T	-0.0711	0.0106	1.95E-11	6.86E-05	44.99118904
rs753270	C	T	0.0528	0.0079	2.70E-11	6.81E-05	44.66976446
rs7561798	G	A	0.04	0.0072	2.79E-08	4.71E-05	30.86419753
rs7572970	G	A	0.059	0.0087	1.39E-11	7.01E-05	45.99022328
rs7607777	T	G	-0.137	0.0125	9.40E-28	0.000183172	120.1216
rs7674212	T	G	-0.0465	0.0075	6.18E-10	5.86E-05	38.44
rs7685296	T	C	-0.0511	0.0081	2.32E-10	6.07E-05	39.79896357
rs7729395	T	C	0.1373	0.016	1.10E-17	0.000112298	73.63785156
rs7786095	G	A	-0.0743	0.0129	9.64E-09	5.06E-05	33.174028
rs780094	C	T	0.0692	0.0074	5.16E-21	0.000133355	87.4477721
rs7845219	C	T	-0.0422	0.0072	4.54E-09	5.24E-05	34.35262346

rs7903146	T	C	0.3059	0.0077	1.00E-200	0.002401331	1578.256198
rs7929543	C	A	0.0828	0.0138	2.20E-09	5.49E-05	36
rs7955901	T	C	-0.0444	0.0072	7.16E-10	5.80E-05	38.02777778
rs8068804	A	G	0.0587	0.0078	4.41E-14	8.64E-05	56.63527285
rs8108269	G	T	0.0644	0.0079	3.11E-16	0.000101343	66.45345297
rs825476	T	C	0.0524	0.0073	6.80E-13	7.86E-05	51.52486395
rs840967	A	C	-0.0497	0.008	5.44E-10	5.89E-05	38.59515625
rs849135	A	G	-0.0999	0.0072	1.04E-43	0.000293533	192.515625
rs853974	C	T	-0.0601	0.0088	7.86E-12	7.11E-05	46.64269112
rs9369425	A	G	-0.0546	0.0085	1.13E-10	6.29E-05	41.2617301
rs963740	T	A	-0.0479	0.0086	2.23E-08	4.73E-05	31.02230936
rs9844972	C	G	0.0956	0.0148	1.03E-10	6.36E-05	41.72461651
rs9894220	G	A	-0.0585	0.0079	1.52E-13	8.36E-05	54.83496235
rs993380	G	A	-0.0507	0.0081	4.59E-10	5.98E-05	39.17832647
rs9940149	A	G	-0.058	0.0095	9.29E-10	5.68E-05	37.27423823

Supplementary Table S3. Genetic instruments used in MR analysis of HbA1c and preeclampsia

SNP	EA	OA	Beta	SE	p	R2	F-statistic
rs10151436	T	A	-0.013	0.0021	3.85E-11	2.61E-04	38.32199546
rs10169706	T	C	0.026	0.0046	1.48E-08	2.18E-04	31.94706994
rs10231021	A	T	0.0089	0.0013	8.69E-14	3.19E-04	46.86982249
rs10405535	G	A	-0.0122	0.0016	6.47E-14	3.96E-04	58.140625
rs10811661	C	T	-0.0128	0.0017	1.74E-14	3.86E-04	56.69204152
rs10830963	G	C	0.0197	0.0015	1.54E-36	1.17E-03	172.4844444
rs11248914	C	T	-0.0114	0.0014	1.42E-14	4.51E-04	66.30612245
rs11257655	T	C	0.011	0.0016	1.91E-13	0.000321861	47.265625
rs11558471	G	A	-0.0151	0.0014	3.38E-25	7.92E-04	116.3316327
rs11643024	G	A	-0.0084	0.0015	7.97E-10	0.000213573	31.36
rs11720108	T	C	-0.0129	0.0015	2.95E-18	0.000503547	73.96
rs117233107	A	G	-0.047	0.0072	8.45E-11	0.00029018	42.61188272
rs1175549	C	A	-0.0098	0.0015	7.13E-13	2.91E-04	42.68444444
rs12491937	G	A	-0.009	0.0013	1.42E-13	3.26E-04	47.92899408
rs12612492	T	C	0.0188	0.0019	1.88E-26	0.000666471	97.90581717
rs1278769	G	A	0.0091	0.0015	5.51E-12	0.000250642	36.80444444
rs13089972	A	T	0.0111	0.0014	1.87E-15	4.28E-04	62.8622449
rs13134327	A	G	0.0144	0.0014	2.81E-26	7.20E-04	105.7959184
rs13234131	G	A	0.0113	0.002	2.06E-09	2.17E-04	31.9225
rs13389076	A	G	0.0332	0.0038	3.04E-18	5.20E-04	76.33240997
rs13419763	T	C	0.008	0.0014	5.48E-09	2.22E-04	32.65306122
rs151165	A	T	0.0079	0.0014	2.04E-09	2.17E-04	31.84183673
rs1535464	A	G	-0.0086	0.0017	1.11E-08	1.74E-04	25.5916955
rs174559	A	G	-0.0106	0.0014	3.31E-13	3.90E-04	57.32653061
rs17476364	C	T	-0.0858	0.0023	1.00E-200	9.39E-03	1391.614367
rs17533945	C	T	0.0128	0.0014	1.62E-23	5.69E-04	83.59183673
rs1800562	A	G	-0.0383	0.0027	2.33E-50	1.37E-03	201.2194787
rs2001945	C	G	0.0069	0.0013	6.18E-10	1.92E-04	28.17159763
rs2015803	T	C	0.0112	0.0016	2.28E-12	0.000333667	49
rs2375278	G	A	-0.0112	0.0017	1.05E-11	0.000295578	43.40484429
rs2613522	G	A	0.027	0.0021	9.34E-39	0.001124766	165.3061224
rs267738	G	T	-0.0109	0.0016	1.14E-11	3.16E-04	46.41015625
rs2908277	A	G	0.0166	0.002	1.29E-18	4.69E-04	68.89
rs2971670	T	C	0.0316	0.0017	5.1004E-88	0.002348105	345.5224913
rs340882	G	C	0.0084	0.0013	1.48E-10	2.84E-04	41.75147929
rs360147	T	C	-0.0086	0.0015	2.08E-09	2.24E-04	32.87111111
rs3778321	A	G	-0.0106	0.0016	4.18E-11	2.99E-04	43.890625
rs3829109	A	G	-0.0086	0.0015	2.68E-08	2.24E-04	32.87111111
rs452306	T	C	-0.0098	0.0014	5.51E-13	3.34E-04	49
rs4727979	C	A	-0.0121	0.0024	4.61E-08	1.73E-04	25.41840278
rs4737009	A	G	0.0228	0.0015	8.29E-56	1.57E-03	231.04
rs4760682	A	C	0.0164	0.0018	3.20E-20	0.000565144	83.01234568
rs4980325	T	G	0.0108	0.0014	4.70E-14	4.05E-04	59.51020408
rs560887	C	T	0.0307	0.0014	5.55E-122	0.003264845	480.8622449
rs608793	T	C	0.0065	0.0013	4.54E-08	0.000170266	25
rs61750929	T	C	-0.0284	0.0029	9.49E-24	0.000652859	95.90487515

rs6474359	C	T	-0.0427	0.0038	1.91E-33	8.59E-04	126.2666205
rs651007	T	C	0.0108	0.0015	3.28E-15	3.53E-04	51.84
rs6804915	A	C	-0.0108	0.0014	2.76E-16	0.000405208	59.51020408
rs6877043	C	T	-0.0085	0.0014	1.99E-10	0.000251035	36.8622449
rs7042939	G	A	-0.0102	0.0013	1.50E-15	4.19E-04	61.56213018
rs7127313	T	C	0.0066	0.0013	4.85E-08	1.76E-04	25.77514793
rs7198799	T	C	0.0083	0.0014	4.76E-09	2.39E-04	35.14795918
rs737092	C	T	0.0073	0.0013	7.57E-09	2.15E-04	31.53254438
rs7547793	C	A	0.0118	0.0021	6.61E-09	2.15E-04	31.57369615
rs7861647	T	C	0.0128	0.0016	4.50E-14	4.36E-04	64
rs79403657	G	C	0.009	0.0017	2.03E-08	1.91E-04	28.02768166
rs8138197	A	G	-0.0073	0.0014	3.54E-08	1.85E-04	27.18877551
rs837763	T	C	0.0176	0.0013	5.20E-38	1.25E-03	183.2899408
rs855791	G	A	-0.0188	0.0013	1.34E-56	1.42E-03	209.1360947
rs857725	G	T	0.0208	0.0014	5.43E-55	1.50E-03	220.7346939
rs9376090	C	T	-0.0247	0.0014	1.90E-62	2.12E-03	311.2704082
rs9818758	A	G	0.0131	0.0017	1.49E-13	0.000404326	59.38062284
rs9909940	T	C	0.0322	0.0014	1.43E-116	0.003590506	529
rs9914988	A	G	0.0125	0.0016	4.66E-17	0.000415587	61.03515625

Supplementary Table S4. Genetic instruments used in MR analysis of FI and preeclampsia

SNP	EA	OA	Beta	SE	p	R2	F-statistic
rs10050393	C	T	-0.009	0.0019	4.84E-08	1.49E-04	22.43767313
rs10865959	C	G	0.0138	0.0022	1.99E-08	2.60E-04	39.34710744
rs116141873	T	G	0.0428	0.0059	1.42E-11	3.48E-04	52.62395863
rs11708067	G	A	0.0135	0.0023	1.30E-09	2.28E-04	34.45179584
rs11727676	C	T	0.0203	0.0039	2.90E-08	1.79E-04	27.09335963
rs118164457	C	T	0.0345	0.0057	3.86E-10	2.43E-04	36.63434903
rs1206760	A	G	-0.0112	0.0019	8.82E-10	2.30E-04	34.74792244
rs12454712	C	T	-0.0142	0.0025	1.78E-09	0.000213597	32.2624
rs1260326	C	T	0.0231	0.0019	8.42E-38	9.78E-04	147.8144044
rs13258890	C	T	-0.0128	0.0025	2.77E-08	0.000173563	26.2144
rs13389219	T	C	-0.0199	0.0019	5.84E-28	0.000725897	109.6980609
rs1351394	C	T	0.0111	0.0018	2.71E-09	0.000251758	38.02777778
rs1474696	G	A	0.0147	0.0018	3.02E-16	4.41E-04	66.69444444
rs17036126	T	C	0.0209	0.003	1.28E-10	3.21E-04	48.53444444
rs17331151	T	C	-0.0162	0.0031	1.52E-08	0.000180809	27.30905307
rs2108349	A	G	-0.0115	0.002	1.13E-08	0.000218893	33.0625
rs2780215	G	A	-0.0392	0.0063	1.06E-09	2.56E-04	38.71604938
rs2845885	T	C	-0.0204	0.0039	1.18E-08	1.81E-04	27.36094675
rs35000407	G	T	-0.0258	0.0028	1.50E-21	5.62E-04	84.90306122
rs3775380	G	A	0.0119	0.0018	1.48E-11	2.89E-04	43.70679012
rs459193	G	A	0.0181	0.0021	1.12E-18	4.92E-04	74.28798186
rs4865796	A	G	0.0165	0.002	7.33E-17	4.51E-04	68.0625
rs5017305	T	A	-0.0137	0.0026	1.07E-08	1.84E-04	27.7647929
rs62271373	A	T	0.0256	0.0048	1.60E-08	1.88E-04	28.44444444
rs6487237	A	C	0.0154	0.0026	4.68E-09	2.32E-04	35.08284024
rs6855363	C	T	-0.0125	0.002	4.04E-08	2.59E-04	39.0625
rs7012814	A	G	-0.0219	0.0019	8.34E-30	8.79E-04	132.8559557
rs7133378	A	G	-0.0127	0.002	6.00E-11	2.67E-04	40.3225
rs73013411	A	C	-0.018	0.0032	2.08E-08	0.000209481	31.640625
rs731839	A	G	-0.0121	0.0019	3.86E-11	0.000268496	40.5567867
rs75179845	C	T	0.0216	0.0035	6.05E-11	0.000252147	38.08653061
rs860598	A	G	0.0177	0.0025	6.88E-12	3.32E-04	50.1264
rs9884482	C	T	0.0125	0.0019	2.88E-11	2.87E-04	43.28254848

Supplementary Table S5. Genetic instruments used in MR analysis of BMI and preeclampsia

SNP	EA	OA	Beta	SE	p	R2	F-statistic
rs10009336	T	C	-0.014	0.0022	2.20E-10	5.94E-05	40.49586777
rs1006896	C	A	-0.0234	0.0027	5.50E-18	1.10E-04	75.11111111
rs10132280	A	C	-0.0223	0.0018	5.60E-35	2.25E-04	153.4845679
rs10169594	C	T	0.0121	0.0018	2.00E-11	6.63E-05	45.1882716
rs10182181	G	A	0.0325	0.0016	6.70E-90	6.05E-04	412.5976563
rs10192119	G	T	0.0166	0.0022	3.00E-14	8.36E-05	56.9338843
rs10197031	C	T	0.0166	0.0019	1.90E-18	1.12E-04	76.33240997
rs10243319	C	T	-0.0107	0.0018	1.20E-09	5.18655E-05	35.33641975
rs10247983	A	G	0.0201	0.0033	1.70E-09	5.45E-05	37.09917355
rs10248136	T	C	-0.0097	0.0017	2.00E-08	4.77863E-05	32.55709343
rs10269783	A	G	0.0133	0.0017	1.40E-15	8.98349E-05	61.20761246
rs10478110	C	A	0.01	0.0017	9.60E-09	5.07877E-05	34.60207612
rs1048932	A	C	-0.016	0.0017	3.80E-22	1.30E-04	88.58131488
rs10492229	T	C	0.0142	0.0019	7.70E-14	8.20E-05	55.85595568
rs10510419	T	G	-0.0177	0.0023	2.20E-14	8.69224E-05	59.22306238
rs10518694	A	C	0.0146	0.0025	3.30E-09	5.00591E-05	34.1056
rs1064213	A	G	0.012	0.0017	2.40E-12	7.31E-05	49.82698962
rs10733051	G	A	-0.0097	0.0016	2.90E-09	5.39E-05	36.75390625
rs10742752	C	T	0.0124	0.0017	1.10E-13	7.81E-05	53.20415225
rs10747488	A	C	-0.0123	0.002	1.20E-09	5.55E-05	37.8225
rs1075901	C	T	0.0121	0.0016	1.20E-13	8.39E-05	57.19140625
rs10768994	C	T	-0.0114	0.0017	6.40E-12	6.60E-05	44.96885813
rs10795422	G	A	0.0139	0.0019	9.30E-14	7.86E-05	53.52077562
rs10811871	G	A	-0.0108	0.0018	1.60E-09	5.28E-05	36
rs10832778	G	C	0.0125	0.0017	1.30E-13	7.94E-05	54.06574394
rs10858334	G	C	0.0143	0.0026	2.70E-08	4.44E-05	30.25
rs10867256	T	C	-0.0118	0.0017	8.70E-12	7.07E-05	48.1799308
rs10878946	T	C	-0.0141	0.0019	3.60E-13	8.08E-05	55.07202216
rs10887578	C	G	0.0128	0.0017	1.60E-13	8.32079E-05	56.69204152
rs10914462	G	A	-0.0112	0.0017	1.50E-10	6.37073E-05	43.40484429
rs10915840	A	G	-0.0118	0.0019	1.30E-09	5.66123E-05	38.57063712
rs10920678	G	A	-0.0155	0.0016	1.50E-21	1.38E-04	93.84765625
rs10938397	G	A	0.0324	0.0016	3.40E-86	6.02E-04	410.0625
rs10942267	G	A	-0.0156	0.0019	3.90E-17	9.89E-05	67.41274238
rs10953740	G	A	-0.0153	0.0017	1.00E-18	1.19E-04	81
rs10962550	C	G	0.0182	0.0022	6.20E-16	1.00E-04	68.43801653
rs10968114	C	A	-0.0113	0.0017	6.10E-11	6.48E-05	44.183391
rs10971709	T	C	0.0132	0.0021	6.20E-10	5.80E-05	39.51020408
rs10984756	G	C	0.0174	0.0029	1.10E-09	5.28E-05	36
rs11030618	T	C	0.011	0.0017	2.40E-10	6.14525E-05	41.86851211
rs11084553	G	A	-0.021	0.0024	1.80E-18	0.000112369	76.5625
rs11105839	A	T	-0.0109	0.0017	1.10E-10	6.03E-05	41.11072664
rs11118308	G	A	-0.0101	0.0016	4.80E-10	5.85E-05	39.84765625
rs1112613	A	G	-0.0133	0.0023	3.40E-09	4.90801E-05	33.43856333
rs11150911	C	A	-0.0133	0.0018	4.70E-13	8.01E-05	54.59567901
rs11165643	T	C	0.0206	0.0017	1.40E-35	2.15E-04	146.8373702

rs11170468	C	A	-0.0123	0.0019	1.90E-10	6.15E-05	41.90858726
rs11173522	A	C	0.0128	0.0021	1.10E-09	5.45E-05	37.15192744
rs11185111	A	G	-0.0129	0.0019	7.70E-12	6.77E-05	46.09695291
rs11251352	G	A	0.0109	0.0018	7.00E-10	5.38224E-05	36.66975309
rs1144387	C	G	0.0098	0.0017	1.60E-08	4.88E-05	33.23183391
rs11496125	T	C	0.0169	0.0017	3.00E-22	1.45E-04	98.82698962
rs11505821	T	A	0.0311	0.0035	2.70E-19	1.16E-04	78.95591837
rs11538	G	A	0.0135	0.0023	3.30E-09	5.06E-05	34.45179584
rs1158805	A	C	-0.0137	0.0018	1.20E-14	8.50E-05	57.92901235
rs11609659	C	T	-0.0154	0.002	2.20E-14	8.70207E-05	59.29
rs11611246	T	G	0.024	0.002	5.00E-32	2.11E-04	144
rs11615578	T	C	0.013	0.002	8.10E-11	6.20E-05	42.25
rs11672660	T	C	-0.034	0.0021	1.70E-60	0.000384619	262.1315193
rs11713193	A	G	0.0239	0.0017	2.40E-44	2.90E-04	197.650519
rs11736228	T	A	-0.0139	0.002	4.10E-12	7.09E-05	48.3025
rs11738695	A	C	0.0097	0.0017	2.00E-08	4.78E-05	32.55709343
rs11739877	T	C	0.0117	0.0018	6.60E-11	6.20E-05	42.25
rs11781699	C	T	0.0132	0.0021	3.10E-10	5.80E-05	39.51020408
rs11855853	T	C	-0.0145	0.002	2.40E-13	7.71474E-05	52.5625
rs1187352	C	T	0.0119	0.0018	6.00E-11	6.41505E-05	43.70679012
rs11880870	G	A	-0.0189	0.0017	1.00E-28	1.81E-04	123.6020761
rs11889536	G	A	-0.0189	0.0024	6.40E-15	9.10E-05	62.015625
rs11908637	A	G	-0.012	0.0021	4.90E-09	4.79E-05	32.65306122
rs11945861	A	G	-0.0148	0.002	5.00E-13	8.04E-05	54.76
rs11951673	T	C	-0.0123	0.0017	1.10E-13	7.68E-05	52.34948097
rs12041258	C	T	-0.0146	0.002	9.50E-13	7.82E-05	53.29
rs12049202	T	C	0.024	0.0022	1.00E-28	1.75E-04	119.0082645
rs12098284	T	C	0.0178	0.0026	1.80E-11	6.88E-05	46.86982249
rs12150665	C	T	-0.0162	0.0017	1.60E-22	0.000133276	90.80968858
rs1218822	A	G	0.0168	0.0017	1.90E-22	1.43E-04	97.66089965
rs12299814	A	C	-0.0157	0.002	5.20E-15	9.04E-05	61.6225
rs12328930	C	T	0.0098	0.0017	1.80E-08	4.88E-05	33.23183391
rs12334877	A	G	-0.0144	0.0022	7.70E-11	6.28827E-05	42.84297521
rs12364470	G	T	0.0178	0.0022	1.10E-15	9.61E-05	65.46280992
rs12369179	T	C	-0.0359	0.0031	2.50E-31	0.000196815	134.1113424
rs12416812	A	G	0.0111	0.0016	6.10E-12	7.06E-05	48.12890625
rs1241986	A	G	-0.0139	0.0024	1.10E-08	4.92339E-05	33.54340278
rs12422552	C	G	-0.0134	0.002	1.60E-11	6.59E-05	44.89
rs12429545	A	G	0.0316	0.0025	9.60E-38	2.34E-04	159.7696
rs12448257	A	G	0.0184	0.002	8.10E-20	1.24E-04	84.64
rs12546578	A	T	0.0146	0.002	1.00E-13	7.82E-05	53.29
rs12564992	G	A	0.0196	0.0026	5.30E-14	8.34E-05	56.82840237
rs12593036	G	A	-0.0154	0.0019	3.80E-16	9.64209E-05	65.69529086
rs12602912	T	C	0.0176	0.0021	9.90E-18	1.03E-04	70.24036281
rs1260326	C	T	0.0105	0.0017	3.90E-10	5.60E-05	38.14878893
rs12629015	G	A	-0.0135	0.0023	2.10E-09	5.05672E-05	34.45179584
rs1266874	G	A	0.014	0.0018	9.80E-15	8.88E-05	60.49382716
rs12675063	T	A	0.0156	0.0026	1.30E-09	5.28395E-05	36
rs1268065	A	G	-0.0102	0.0017	1.00E-09	5.28E-05	36

rs12680842	G	A	-0.0133	0.0018	4.40E-14	8.01313E-05	54.59567901
rs12718572	T	C	-0.0117	0.0018	3.00E-11	6.20E-05	42.25
rs12762034	C	T	0.024	0.0032	7.30E-14	8.26E-05	56.25
rs12779328	T	C	0.0105	0.0019	4.50E-08	4.48E-05	30.5401662
rs1285997	G	C	0.0142	0.0019	1.20E-13	8.19809E-05	55.85595568
rs12888545	G	A	0.0136	0.002	9.10E-12	6.79E-05	46.24
rs12888955	A	G	-0.0178	0.0018	1.40E-22	1.44E-04	97.79012346
rs12905439	G	C	-0.0118	0.0018	1.40E-10	6.30769E-05	42.97530864
rs12914489	A	G	0.0165	0.0026	3.80E-10	5.91E-05	40.27366864
rs12922346	C	G	0.0136	0.002	1.00E-11	6.79E-05	46.24
rs12933482	G	A	0.0186	0.0028	4.90E-11	6.48E-05	44.12755102
rs12936083	G	A	0.0139	0.0019	4.10E-13	7.86E-05	53.52077562
rs12939549	G	A	-0.018	0.0016	2.70E-28	1.86E-04	126.5625
rs1296328	C	A	-0.0179	0.0018	4.90E-24	1.45E-04	98.89197531
rs12981256	A	G	0.0142	0.0018	1.10E-15	9.13E-05	62.2345679
rs13021737	G	A	0.0574	0.0021	7.499E-157	0.001095439	747.1111111
rs13047416	G	C	-0.0154	0.0018	2.1999E-17	0.000107431	73.19753086
rs13069244	A	G	0.0187	0.0032	3E-09	5.01234E-05	34.14941406
rs13107325	T	C	0.047	0.0032	1.1E-47	0.000316546	215.7226563
rs13110266	A	G	-0.0117	0.0017	1.9002E-12	6.9522E-05	47.36678201
rs13147390	C	T	0.0103	0.0018	0.00000001	4.80604E-05	32.74382716
rs13174863	G	A	0.0192	0.0023	2.9E-16	0.000102278	69.68620038
rs13184896	T	G	-0.0133	0.0016	3.2999E-16	0.000101414	69.09765625
rs13191362	G	A	-0.0236	0.0025	5.9007E-21	0.000130787	89.1136
rs1321432	C	A	0.0201	0.0018	3.5003E-29	0.000182998	124.6944444
rs13250058	T	G	0.0112	0.0018	2.9E-10	5.68258E-05	38.71604938
rs13263601	C	A	0.0154	0.0018	2.1999E-17	0.000107431	73.19753086
rs1327259	G	A	-0.0155	0.0018	1.6998E-18	0.00010883	74.15123457
rs1330052	G	C	0.0132	0.0018	1.5E-13	7.8931E-05	53.77777778
rs13329567	T	C	-0.0293	0.002	1E-50	0.000314932	214.6225
rs1365466	T	C	-0.0137	0.0019	3.2999E-13	7.63097E-05	51.99168975
rs1371108	A	C	0.0119	0.0018	8.9991E-11	6.41505E-05	43.70679012
rs138289	T	A	-0.0103	0.0017	3.3E-09	5.38806E-05	36.70934256
rs1409818	T	C	0.0201	0.0029	2.4998E-12	7.0509E-05	48.039239
rs1412235	C	G	0.0246	0.0017	6.0007E-45	0.000307268	209.3979239
rs1421334	C	A	-0.0125	0.0018	1E-12	7.0782E-05	48.22530864
rs1430387	C	T	-0.0114	0.0017	5.7996E-11	6.60027E-05	44.96885813
rs1431659	G	A	-0.0196	0.0019	6.0007E-24	0.000156177	106.4155125
rs1436344	C	G	0.0141	0.0017	4.1002E-16	0.000100966	68.79238754
rs1445652	A	G	0.0123	0.0022	4.3E-08	4.588E-05	31.25826446
rs1452075	T	C	0.0141	0.0018	1.2999E-14	9.00602E-05	61.36111111
rs1454687	G	C	-0.0202	0.0017	5.2E-32	0.000207202	141.1903114
rs1465900	C	A	-0.0125	0.002	4.8E-10	5.73342E-05	39.0625
rs1472169	T	C	-0.0139	0.0018	2.8003E-15	8.75236E-05	59.63271605
rs1476322	A	G	0.0101	0.0017	5E-09	5.18085E-05	35.29757785
rs1477199	G	A	0.0228	0.0024	9.3994E-22	0.000132455	90.25
rs1492767	T	C	0.0094	0.0016	0.00000001	5.06609E-05	34.515625
rs1503526	C	T	0.014	0.0017	5.5005E-17	9.95391E-05	67.8200692
rs1521527	C	G	-0.0121	0.0017	3.1003E-12	7.43566E-05	50.66089965

rs1522569	G	T	-0.0164	0.0022	2.9E-13	8.15616E-05	55.57024793
rs1528435	T	C	0.0164	0.0017	9.0991E-23	0.000136587	93.06574394
rs1535660	C	T	-0.0147	0.0025	5.2E-09	5.07471E-05	34.5744
rs1538247	C	T	0.0108	0.0019	0.00000001	4.7424E-05	32.31024931
rs1552893	G	A	-0.0126	0.0019	8.1003E-11	6.45483E-05	43.97783934
rs156201	C	G	0.0123	0.002	5.8E-10	5.55143E-05	37.8225
rs1624134	C	G	0.0101	0.0018	1.1E-08	4.62122E-05	31.4845679
rs1656377	C	T	0.0099	0.0017	1.6E-08	4.97771E-05	33.91349481
rs1681740	C	A	-0.0115	0.0018	1.1E-10	5.99106E-05	40.81790123
rs16849710	G	A	-0.0116	0.0018	6.0007E-11	6.0957E-05	41.5308642
rs16851483	T	G	0.0369	0.0035	3.1996E-26	0.000163127	111.1518367
rs16871902	A	G	0.0125	0.0017	4.6005E-13	7.93536E-05	54.06574394
rs16903285	C	T	0.0331	0.0026	7.5998E-38	0.00023784	162.0724852
rs17001561	A	G	0.0151	0.0023	3.8001E-11	6.3263E-05	43.1020794
rs17014375	G	T	0.0172	0.0025	1.1E-11	6.94745E-05	47.3344
rs17033117	T	C	0.0137	0.0022	8.9E-10	5.6918E-05	38.77892562
rs17056301	C	T	0.0118	0.002	2.4E-09	5.10929E-05	34.81
rs17113297	T	C	0.0166	0.0021	2.0999E-15	9.171E-05	62.48526077
rs17119937	C	T	0.0212	0.0036	5.6E-09	5.09007E-05	34.67901235
rs17207196	T	C	-0.0221	0.0018	2.0999E-35	0.000221219	150.7438272
rs17238110	G	A	-0.0353	0.005	1.9999E-12	7.31571E-05	49.8436
rs17311369	T	C	-0.0104	0.0019	3.1E-08	4.39764E-05	29.96121884
rs17399237	C	T	-0.0129	0.0017	6.7004E-14	8.4513E-05	57.58131488
rs17405819	C	T	-0.0215	0.0018	4.3003E-33	0.000209373	142.6697531
rs17424296	A	G	-0.0108	0.0018	2.4E-09	5.28395E-05	36
rs17425707	C	T	0.0167	0.0028	4.4E-09	5.22123E-05	35.57270408
rs17446257	A	G	0.0153	0.0026	2.9E-09	5.08268E-05	34.62869822
rs17499593	G	C	0.0125	0.0022	1.1E-08	4.73841E-05	32.28305785
rs17513613	C	T	0.0186	0.0018	3.6E-26	0.000156708	106.7777778
rs175165	G	T	-0.0103	0.0018	5.2E-09	4.80604E-05	32.74382716
rs17535749	A	G	0.015	0.0027	2.5E-08	4.53017E-05	30.86419753
rs17551974	A	C	-0.0141	0.0022	1.9E-10	6.029E-05	41.07644628
rs17663412	A	C	0.0157	0.0027	6.1E-09	4.96283E-05	33.81207133
rs17710386	C	T	0.0126	0.0018	1E-12	7.1919E-05	49
rs17789218	C	T	0.013	0.0019	7.3995E-12	6.87114E-05	46.81440443
rs17806379	T	C	-0.0258	0.0022	1.5E-30	0.00020183	137.5289256
rs1784460	A	T	0.0132	0.0018	8.9991E-14	7.8931E-05	53.77777778
rs1804528	A	G	0.0109	0.002	3E-08	4.35966E-05	29.7025
rs1830074	C	T	0.0115	0.0019	1.4E-09	5.37705E-05	36.63434903
rs1836303	G	A	0.0116	0.0018	5.3003E-11	6.0957E-05	41.5308642
rs1863652	A	G	-0.0115	0.0018	1.4E-10	5.99106E-05	40.81790123
rs1884389	T	C	-0.0103	0.0017	4E-09	5.38806E-05	36.70934256
rs1885728	A	G	0.0108	0.0019	0.00000001	4.7424E-05	32.31024931
rs1891216	G	T	0.0107	0.0018	2.4E-09	5.18655E-05	35.33641975
rs1896767	A	G	-0.0109	0.0017	2.4E-10	6.03403E-05	41.11072664
rs189843	C	G	-0.0098	0.0017	1.7E-08	4.87766E-05	33.23183391
rs1928295	C	T	-0.0141	0.0016	5.4001E-18	0.00011398	77.66015625
rs1937683	T	C	0.0109	0.0018	3.2E-09	5.38224E-05	36.66975309
rs1948080	G	T	-0.0137	0.0018	1.1E-14	8.50233E-05	57.92901235

rs1982725	T	C	0.0097	0.0017	3.3E-08	4.77863E-05	32.55709343
rs1993709	G	A	0.0331	0.0021	1.9002E-57	0.000364534	248.4376417
rs2007231	T	C	-0.0104	0.0018	5.2E-09	4.89981E-05	33.38271605
rs200810	C	T	-0.0136	0.0017	5.5005E-16	9.3933E-05	64
rs2009416	T	C	-0.0121	0.0018	1.1E-11	6.63248E-05	45.1882716
rs2033529	G	A	0.0205	0.0018	1.9002E-30	0.000190353	129.7067901
rs2051559	C	T	0.0176	0.0026	5.0004E-12	6.72556E-05	45.82248521
rs2065418	G	T	-0.0166	0.0018	3.6E-20	0.000124823	85.04938272
rs208015	C	T	-0.0356	0.0034	1.3999E-25	0.000160898	109.633218
rs2124499	C	G	-0.0123	0.0017	3.4002E-13	7.68348E-05	52.34948097
rs2143253	A	G	-0.0188	0.0026	1.1E-12	7.67387E-05	52.28402367
rs215634	G	A	-0.0152	0.0018	2.6002E-17	0.000104659	71.30864198
rs2162524	C	T	0.0155	0.0018	4.1002E-17	0.00010883	74.15123457
rs2163188	C	G	0.0131	0.0017	1.9999E-14	8.71537E-05	59.38062284
rs2174307	C	G	0.0121	0.0017	4.9E-12	7.43566E-05	50.66089965
rs217671	G	A	0.0144	0.0019	1.2999E-13	8.43063E-05	57.44044321
rs2228213	A	G	-0.0139	0.0017	4.6005E-16	9.81224E-05	66.85467128
rs2235564	T	C	0.0131	0.0018	3.7E-13	7.77397E-05	52.96604938
rs2246012	C	T	0.0158	0.0022	3.1003E-13	7.57033E-05	51.5785124
rs226000	T	C	0.0119	0.0022	3.6E-08	4.29446E-05	29.25826446
rs2283093	T	C	0.0127	0.0021	3.1E-09	5.36815E-05	36.57369615
rs2284746	G	C	-0.0104	0.0017	1.4E-09	5.49318E-05	37.42560554
rs2285178	C	T	0.0112	0.0019	9.4001E-09	5.10018E-05	34.74792244
rs2306537	G	A	0.0133	0.0019	8.6996E-13	7.1919E-05	49
rs2307111	C	T	-0.0265	0.0016	1.5999E-58	0.000402491	274.3164063
rs2317299	C	T	-0.0106	0.0017	1.3E-09	5.70648E-05	38.87889273
rs2325036	C	A	-0.0181	0.0017	3.6E-27	0.000166366	113.3598616
rs2357760	A	G	0.0145	0.0017	6.7999E-17	0.000106775	72.75086505
rs2361988	C	T	-0.0155	0.002	5.2E-15	8.81544E-05	60.0625
rs2365389	T	C	-0.0174	0.0017	1.2999E-25	0.000153749	104.7612457
rs2367112	G	T	-0.0119	0.0016	2.2999E-13	8.11891E-05	55.31640625
rs2411182	A	G	0.0123	0.0019	7.7002E-11	6.15113E-05	41.90858726
rs2423668	C	T	-0.0105	0.0019	2.8E-08	4.48261E-05	30.5401662
rs2425840	C	A	0.0119	0.0018	1.5999E-11	6.41505E-05	43.70679012
rs2429150	C	A	0.0111	0.0018	2.7E-10	5.58156E-05	38.02777778
rs2479958	G	A	-0.0154	0.0018	1.5E-17	0.000107431	73.19753086
rs2481665	C	T	-0.0161	0.0016	7.1995E-23	0.000148602	101.2539063
rs2543132	C	G	0.0146	0.0022	5.0004E-11	6.46415E-05	44.04132231
rs2600226	T	C	-0.0116	0.0019	3.7E-10	5.47096E-05	37.27423823
rs2605603	A	G	-0.0103	0.0016	2.5E-10	6.08257E-05	41.44140625
rs2608703	A	C	0.0142	0.0017	1.9002E-16	0.000102403	69.7716263
rs262130	T	C	0.0127	0.0023	1.8E-08	4.47519E-05	30.48960302
rs2643452	A	T	0.0136	0.0017	4.7E-15	9.3933E-05	64
rs2693826	A	G	-0.0137	0.0017	1.9999E-15	9.53193E-05	64.94463668
rs2694047	G	A	0.0188	0.002	3.9003E-21	0.000129682	88.36
rs273504	G	A	0.0153	0.0018	4.4005E-18	0.00010604	72.25
rs2744974	T	C	0.0249	0.0018	1.3999E-45	0.000280809	191.3611111
rs2791653	G	A	-0.0141	0.0019	1.2999E-13	8.08304E-05	55.07202216
rs2820311	G	A	0.0235	0.0018	4.1002E-38	0.000250127	170.4475309

rs2832283	A	G	0.0115	0.002	5.8E-09	4.85281E-05	33.0625
rs2836964	C	T	-0.011	0.0018	1.3E-09	5.48145E-05	37.34567901
rs2861683	C	A	-0.0144	0.0017	1.2999E-16	0.000105308	71.75086505
rs287104	A	G	0.0115	0.0017	4.4005E-11	6.71657E-05	45.76124567
rs2875762	C	G	0.0139	0.002	1.2001E-11	7.08953E-05	48.3025
rs2907948	A	G	-0.0141	0.0019	1.2999E-13	8.08304E-05	55.07202216
rs2943465	C	T	0.0248	0.0039	2E-10	5.93509E-05	40.4365549
rs294704	T	G	-0.0113	0.0019	4E-09	5.19166E-05	35.37119114
rs3007105	T	C	0.0142	0.0017	1.1E-17	0.000102403	69.7716263
rs326896	T	C	-0.0128	0.0018	2.8003E-13	7.42201E-05	50.56790123
rs331966	C	A	0.0112	0.0018	3.2E-10	5.68258E-05	38.71604938
rs33500	T	C	-0.0167	0.0022	4.3003E-14	8.45726E-05	57.62190083
rs339991	G	A	0.0124	0.0018	1.2001E-12	6.96541E-05	47.45679012
rs349088	A	C	-0.0128	0.0017	1.8001E-13	8.32079E-05	56.69204152
rs355777	C	G	0.0153	0.0017	1.3999E-18	0.000118881	81
rs3732084	C	T	0.0107	0.0018	1.1E-09	5.18655E-05	35.33641975
rs3736485	G	A	-0.0134	0.0016	2.4998E-16	0.000102945	70.140625
rs3749897	T	C	0.0122	0.0018	8.4004E-12	6.74255E-05	45.9382716
rs3754963	T	A	-0.0123	0.002	3.3E-10	5.55143E-05	37.8225
rs3764835	A	G	-0.0141	0.0024	3.1E-09	5.06609E-05	34.515625
rs3772882	A	C	0.0127	0.0018	6.5993E-13	7.3065E-05	49.7808642
rs3800229	T	G	0.0175	0.0018	1.3999E-22	0.000138723	94.52160494
rs3800637	C	T	0.0115	0.0018	5.1E-10	5.99106E-05	40.81790123
rs3806114	A	G	-0.0113	0.0018	3.4E-10	5.7845E-05	39.41049383
rs3806572	A	G	-0.0145	0.0019	1.5999E-14	8.54812E-05	58.24099723
rs3807645	A	G	-0.0166	0.0021	2.3999E-15	9.171E-05	62.48526077
rs380857	A	C	-0.0151	0.0027	3.6E-08	4.59077E-05	31.27709191
rs3814883	T	C	0.0232	0.0017	1.1E-40	0.000273299	186.2422145
rs3828783	A	G	-0.0165	0.0021	5.6002E-15	9.06085E-05	61.73469388
rs3829849	T	C	0.0098	0.0017	5.9E-09	4.87766E-05	33.23183391
rs38314	A	G	-0.012	0.0017	4.7E-12	7.31327E-05	49.82698962
rs3844598	G	A	0.0095	0.0017	3.8E-08	4.58362E-05	31.2283737
rs3904244	A	T	0.0155	0.0025	4.3E-10	5.64206E-05	38.44
rs391300	C	T	-0.0119	0.0017	3.1003E-12	7.1919E-05	49
rs3935648	G	C	-0.0125	0.0022	6.8E-09	4.73841E-05	32.28305785
rs3977755	T	C	-0.0135	0.0019	5.9007E-13	7.40981E-05	50.48476454
rs40067	A	G	-0.0266	0.0023	7.1007E-30	0.000196291	133.7542533
rs4012234	G	T	0.0141	0.0018	9.8992E-16	9.00602E-05	61.36111111
rs4072917	A	G	0.0115	0.0018	6.8992E-11	5.99106E-05	40.81790123
rs4148155	G	A	-0.0188	0.0026	5.0004E-13	7.67387E-05	52.28402367
rs4148866	T	C	0.0098	0.0018	0.00000004	4.35078E-05	29.64197531
rs4237643	G	T	-0.0223	0.0019	4.3003E-33	0.000202159	137.7534626
rs427943	C	A	0.017	0.0017	7.2996E-23	0.000146762	100
rs429343	G	A	-0.015	0.0017	6.7999E-18	0.000114265	77.85467128
rs4307239	G	A	0.0115	0.0017	3.9003E-11	6.71657E-05	45.76124567
rs4310573	T	C	0.0116	0.0021	3.5E-08	4.47854E-05	30.51247166
rs4358081	C	A	0.0097	0.0017	1.5E-08	4.77863E-05	32.55709343
rs4430672	C	T	-0.0127	0.0022	3.9E-09	4.89125E-05	33.32438017
rs4482463	A	C	-0.0331	0.0033	2.8003E-23	0.000147653	100.6069789

rs4495304	C	T	-0.0194	0.0033	5E-09	5.07262E-05	34.56014692
rs4516268	A	C	-0.0217	0.0021	5.2E-25	0.000156708	106.7777778
rs4518345	A	G	-0.0117	0.0019	1E-09	5.56569E-05	37.91966759
rs4556997	A	C	0.0197	0.0024	6.9008E-17	9.88885E-05	67.37673611
rs4589691	G	C	0.0141	0.0024	4.7E-09	5.06609E-05	34.515625
rs4639527	G	A	0.0172	0.0019	3.2999E-20	0.000120275	81.9501385
rs4653017	T	C	0.0122	0.0018	4.4999E-11	6.74255E-05	45.9382716
rs4660443	T	C	0.0164	0.0021	6.7999E-15	8.95136E-05	60.98866213
rs4671328	G	T	-0.0219	0.0017	2.1999E-36	0.000243536	165.9550173
rs4722398	T	C	0.0158	0.0025	3.6E-10	5.86256E-05	39.9424
rs4740619	C	T	-0.0186	0.0016	2.2999E-30	0.000198326	135.140625
rs4757144	A	G	0.0169	0.0018	5.6002E-22	0.000129375	88.15123457
rs4783830	A	G	-0.0105	0.0019	2.4E-08	4.48261E-05	30.5401662
rs4786903	G	A	0.0125	0.002	3.5E-10	5.73342E-05	39.0625
rs4800191	C	G	0.0103	0.0017	2.5E-09	5.38806E-05	36.70934256
rs4818225	G	A	0.0117	0.0018	2.3E-10	6.20124E-05	42.25
rs4820408	G	T	-0.0151	0.0017	2.0999E-19	0.000115794	78.89619377
rs4842491	T	C	0.0098	0.0018	0.00000004	4.35078E-05	29.64197531
rs4851029	G	T	0.0121	0.0017	1.6998E-12	7.43566E-05	50.66089965
rs4858193	C	T	-0.0129	0.0019	1.5999E-11	6.76584E-05	46.09695291
rs486359	C	G	0.0112	0.0017	1.5999E-11	6.37073E-05	43.40484429
rs4864201	C	T	-0.0141	0.0017	1.5E-16	0.000100966	68.79238754
rs4880341	T	C	-0.0118	0.0017	1.1E-11	7.07154E-05	48.1799308
rs4906908	G	T	0.0103	0.0017	2.5E-09	5.38806E-05	36.70934256
rs491711	C	A	-0.0115	0.0019	1.1E-09	5.37705E-05	36.63434903
rs4936175	C	T	0.0122	0.0017	1.3999E-12	7.55906E-05	51.5017301
rs4937870	G	A	-0.0109	0.0019	8.8E-09	4.83063E-05	32.91135734
rs4952843	G	A	-0.0131	0.0018	6.7999E-14	7.77397E-05	52.96604938
rs4954638	C	A	-0.0118	0.002	2.9E-09	5.10929E-05	34.81
rs4968656	G	A	0.0116	0.0019	8.1999E-10	5.47096E-05	37.27423823
rs4981693	A	G	0.0206	0.002	6.9008E-24	0.000155699	106.09
rs4986044	T	C	-0.0164	0.0016	3.2999E-23	0.000154191	105.0625
rs538579	C	G	0.0137	0.0019	1.2999E-13	7.63097E-05	51.99168975
rs559231	T	G	0.0135	0.0018	2.3999E-14	8.25592E-05	56.25
rs577525	C	T	0.0166	0.0017	9.7006E-22	0.000139938	95.34948097
rs592483	T	C	-0.0147	0.0017	1.9999E-18	0.000109741	74.7716263
rs6011457	A	T	-0.0116	0.0017	2.7002E-11	6.83388E-05	46.56055363
rs6235	G	C	0.0175	0.0019	1.5E-19	0.000124507	84.83379501
rs6265	T	C	-0.0412	0.0021	1E-86	0.000564663	384.9070295
rs6443750	C	T	0.0148	0.0021	3.1996E-12	7.29008E-05	49.66893424
rs6448587	C	A	-0.0167	0.0023	2.2999E-13	7.73789E-05	52.72022684
rs645040	T	G	0.0171	0.002	2.4998E-18	0.000107291	73.1025
rs6461115	G	A	-0.0144	0.0019	1.2001E-13	8.43063E-05	57.44044321
rs6471941	A	G	0.0156	0.0021	3.1003E-13	8.09943E-05	55.18367347
rs6500208	A	G	0.014	0.002	4.1002E-12	7.1919E-05	49
rs6512302	C	G	0.0142	0.002	2.0999E-12	7.39884E-05	50.41
rs6545714	A	G	-0.0191	0.0017	9.0991E-31	0.000185254	126.2318339
rs6556301	T	G	-0.0111	0.0018	4.1E-10	5.58156E-05	38.02777778
rs6561943	T	C	0.0119	0.0019	4.2E-10	5.75759E-05	39.22714681

rs657452	G	A	-0.0188	0.0017	7.1995E-29	0.000179481	122.2975779
rs6587552	G	A	-0.0173	0.002	1.5999E-17	0.000109815	74.8225
rs6591407	A	C	-0.0118	0.0021	1.9E-08	4.6343E-05	31.57369615
rs6593688	G	A	0.0137	0.0018	8.6E-15	8.50233E-05	57.92901235
rs6595205	G	C	-0.0114	0.0016	1.9999E-12	7.45103E-05	50.765625
rs6673081	C	T	-0.01	0.0018	1.8E-08	4.53017E-05	30.86419753
rs6692586	G	A	-0.0192	0.0023	1.1E-16	0.000102278	69.68620038
rs6712	C	G	0.0138	0.0025	4.4E-08	4.47237E-05	30.4704
rs6764533	A	G	0.0116	0.0018	1.4E-10	6.0957E-05	41.5308642
rs6772756	G	A	-0.0104	0.0019	0.00000004	4.39764E-05	29.96121884
rs6804842	G	A	0.0156	0.0017	3.6E-21	0.000123588	84.20761246
rs6815910	A	T	-0.0128	0.0017	1.3999E-13	8.32079E-05	56.69204152
rs6841761	T	G	-0.0131	0.0016	6.4003E-16	9.83872E-05	67.03515625
rs6985109	A	G	-0.0177	0.0017	1.5E-26	0.000159096	108.4048443
rs7025938	G	C	0.0166	0.0019	3.7E-19	0.000112031	76.33240997
rs7037266	A	C	-0.0112	0.0018	3.5E-10	5.68258E-05	38.71604938
rs705217	G	T	-0.0102	0.0018	9.2999E-09	4.71318E-05	32.11111111
rs7084454	A	G	0.0193	0.0019	4.0004E-25	0.000151433	103.1828255
rs709400	G	A	-0.015	0.0017	4.6005E-19	0.000114265	77.85467128
rs7102454	C	T	0.0158	0.0018	2.3999E-18	0.000113083	77.04938272
rs7117238	A	G	-0.0131	0.0022	2.5E-09	5.20419E-05	35.45661157
rs7124681	A	C	0.0263	0.0016	3.1996E-58	0.000396441	270.1914063
rs7138803	A	G	0.03	0.0017	2.2999E-71	0.000456904	311.4186851
rs7144011	T	G	0.0282	0.002	5.2E-47	0.000291736	198.81
rs7148846	G	T	0.0124	0.0022	2.2E-08	4.66291E-05	31.76859504
rs7172627	G	A	0.0117	0.0017	1.1E-11	6.9522E-05	47.36678201
rs7196720	C	T	-0.0129	0.0017	7.2996E-14	8.4513E-05	57.58131488
rs7206608	G	C	0.0132	0.0019	1.2999E-12	7.08417E-05	48.26592798
rs7222349	A	G	0.0115	0.0018	3.3E-10	5.99106E-05	40.81790123
rs7239575	C	T	-0.0202	0.0017	7.3995E-32	0.000207202	141.1903114
rs7318817	T	C	-0.0155	0.0018	2.7002E-18	0.00010883	74.15123457
rs7334078	C	T	-0.0121	0.0019	2.2E-10	5.95273E-05	40.5567867
rs7358465	T	C	0.0103	0.0019	3E-08	4.31348E-05	29.38781163
rs7488867	T	C	-0.0204	0.002	8.4004E-24	0.000152691	104.04
rs7498665	G	A	0.0271	0.0017	5.6002E-60	0.00037287	254.1211073
rs7519259	A	G	0.0125	0.0017	3.8001E-13	7.93536E-05	54.06574394
rs7535528	A	G	-0.0152	0.0018	1.3999E-16	0.000104659	71.30864198
rs754635	G	C	0.0198	0.0027	2.1999E-13	7.8931E-05	53.77777778
rs7550711	T	C	0.0649	0.005	3.1996E-38	0.000247241	168.4804
rs7551507	T	C	-0.0184	0.0016	9.3004E-30	0.000194084	132.25
rs7557796	C	T	-0.016	0.0018	2.2999E-19	0.000115964	79.01234568
rs756717	A	G	-0.0148	0.0017	5.4001E-18	0.000111239	75.79238754
rs7599312	A	G	-0.0186	0.0019	6.9008E-24	0.000140649	95.83379501
rs7615297	G	C	-0.0149	0.0024	5.6999E-10	5.65724E-05	38.54340278
rs7626079	T	C	0.011	0.0018	1.6E-09	5.48145E-05	37.34567901
rs7637852	G	A	-0.0139	0.0019	1.6998E-13	7.85538E-05	53.52077562
rs7640424	T	C	-0.0136	0.0018	2.2999E-14	8.37867E-05	57.08641975
rs765875	T	C	-0.0121	0.0017	2.9999E-12	7.43566E-05	50.66089965
rs7683836	A	G	-0.0114	0.0017	6.2994E-11	6.60027E-05	44.96885813

rs7685048	T	C	-0.0101	0.0017	4.1E-09	5.18085E-05	35.29757785
rs768840	A	G	0.0114	0.0018	2E-10	5.88732E-05	40.11111111
rs7694732	G	A	-0.0099	0.0017	8.7E-09	4.97771E-05	33.91349481
rs7703576	C	T	0.0103	0.0019	4.8E-08	4.31348E-05	29.38781163
rs7704281	A	G	0.0271	0.0041	6.4998E-11	6.41242E-05	43.68887567
rs7715256	T	G	-0.0166	0.0016	2.1999E-24	0.000157974	107.640625
rs7724675	A	G	-0.0119	0.0021	9.4999E-09	4.71318E-05	32.11111111
rs7730004	T	C	0.0148	0.0018	9.0991E-16	9.92234E-05	67.60493827
rs7730898	A	G	0.0168	0.0018	4.4999E-20	0.000127849	87.11111111
rs774246	G	A	0.0153	0.0025	5.4E-10	5.49741E-05	37.4544
rs7761673	A	T	-0.0126	0.0021	1.9E-09	5.28395E-05	36
rs7780752	C	T	0.0139	0.0018	1E-14	8.75236E-05	59.63271605
rs7788008	A	G	-0.0157	0.0017	1.1E-19	0.000125177	85.29065744
rs7811342	C	T	-0.0197	0.0029	1.1E-11	6.77307E-05	46.14625446
rs7819514	A	G	-0.0107	0.0018	5.6999E-09	5.18655E-05	35.33641975
rs7826312	C	T	0.0104	0.0017	4.9E-10	5.49318E-05	37.42560554
rs7844647	C	T	-0.0123	0.0018	2.8003E-11	6.85353E-05	46.69444444
rs7869771	C	A	-0.014	0.0019	4.9E-13	7.9688E-05	54.29362881
rs7871866	C	G	0.0187	0.0024	2.2999E-14	8.91047E-05	60.71006944
rs7899106	G	A	0.0331	0.0037	1E-18	0.000117457	80.02994887
rs7925214	T	C	0.0147	0.0018	4.4005E-17	9.78872E-05	66.69444444
rs7970953	A	G	0.0135	0.0018	9.7994E-14	8.25592E-05	56.25
rs7983065	T	C	-0.0148	0.0017	8.9002E-18	0.000111239	75.79238754
rs7998796	G	A	0.0105	0.0018	1.1E-08	4.99449E-05	34.02777778
rs8027205	G	C	-0.0108	0.0018	1.4E-09	5.28395E-05	36
rs8036040	A	C	0.0109	0.0017	2.7E-10	6.03403E-05	41.11072664
rs806600	G	A	-0.0095	0.0017	3.3E-08	4.58362E-05	31.2283737
rs8071182	A	G	0.0133	0.0022	2.1E-09	5.3643E-05	36.54752066
rs8090983	G	A	0.0118	0.0018	2E-10	6.30769E-05	42.97530864
rs8097672	T	A	0.02	0.0025	8.4004E-16	9.3933E-05	64
rs8097783	A	G	-0.0389	0.0031	7.1995E-36	0.000231076	157.4620187
rs8123881	G	A	0.0196	0.0024	4.4005E-16	9.78872E-05	66.69444444
rs8181823	C	A	0.0127	0.002	4.1E-10	5.91835E-05	40.3225
rs818524	C	T	0.0106	0.0019	3.4E-08	4.56839E-05	31.12465374
rs8192675	C	T	0.0152	0.0018	1.3999E-17	0.000104659	71.30864198
rs825688	T	C	-0.0095	0.0017	4.7E-08	4.58362E-05	31.2283737
rs845084	A	G	0.014	0.002	1.2999E-12	7.1919E-05	49
rs852056	C	T	-0.0128	0.002	1.8E-10	6.01191E-05	40.96
rs865809	G	A	-0.0127	0.002	5.4E-10	5.91835E-05	40.3225
rs872281	T	C	-0.0151	0.0023	4.7E-11	6.3263E-05	43.1020794
rs876605	G	A	-0.0108	0.002	3.4E-08	4.28004E-05	29.16
rs879620	T	C	0.0231	0.0018	5.3003E-38	0.000241687	164.6944444
rs889398	T	C	-0.0196	0.0016	1.2999E-32	0.000220219	150.0625
rs895330	G	C	-0.0201	0.0023	5.5005E-19	0.00011209	76.37240076
rs901630	T	C	-0.0146	0.0017	1.9002E-18	0.000108253	73.75778547
rs902695	A	G	-0.0103	0.0017	2.2E-09	5.38806E-05	36.70934256
rs9294260	A	G	0.0147	0.0016	1.8001E-19	0.000123885	84.41015625
rs9300422	G	A	-0.0103	0.0018	4E-09	4.80604E-05	32.74382716
rs930295	C	A	-0.0211	0.0023	1E-19	0.000123519	84.16068053

rs9304665	A	T	0.0229	0.002	2.9E-29	0.000192401	131.1025
rs934224	T	C	0.0107	0.002	4.7E-08	4.20115E-05	28.6225
rs9362662	G	A	-0.0112	0.0017	1.2E-10	6.37073E-05	43.40484429
rs9367368	C	T	-0.0121	0.0018	1E-11	6.63248E-05	45.1882716
rs9370261	T	C	0.0231	0.0042	3.4E-08	4.44002E-05	30.25
rs9375702	T	C	-0.0115	0.0019	7.9001E-10	5.37705E-05	36.63434903
rs9379827	A	C	-0.0132	0.0019	6.8992E-12	7.08417E-05	48.26592798
rs9408882	A	G	-0.0093	0.0016	1.3E-08	4.95888E-05	33.78515625
rs946824	C	T	-0.0206	0.0026	1.1E-15	9.21354E-05	62.77514793
rs947612	A	G	-0.0116	0.002	5.6E-09	4.93757E-05	33.64
rs9478671	G	A	0.012	0.0021	1.7E-08	4.79272E-05	32.65306122
rs9522285	A	G	0.0127	0.0017	2.4998E-13	8.1913E-05	55.80968858
rs9538162	C	T	-0.0156	0.0018	4.7995E-19	0.000110239	75.11111111
rs9547153	G	A	0.0098	0.0017	8.7E-09	4.87766E-05	33.23183391
rs9571687	A	C	-0.0129	0.0018	2.8003E-12	7.53842E-05	51.36111111
rs9615905	T	C	0.011	0.0017	2.7E-10	6.14525E-05	41.86851211
rs962273	C	T	0.0137	0.0019	2.6002E-13	7.63097E-05	51.99168975
rs9650755	G	A	0.0154	0.002	2.8003E-15	8.70207E-05	59.29
rs977747	G	T	-0.0169	0.0017	1.2999E-24	0.000145041	98.82698962
rs9783858	T	C	0.0091	0.0017	3.3E-08	4.20577E-05	28.65397924
rs9806742	A	G	0.0208	0.0026	1.3999E-15	9.3933E-05	64
rs9816226	T	A	0.0323	0.0021	1.5999E-52	0.000347132	236.5736961
rs9845966	G	T	-0.0105	0.0017	2.5E-10	5.59932E-05	38.14878893
rs987237	G	A	0.0409	0.0021	9.3004E-84	0.000556474	379.3219955
rs9926784	C	T	-0.0258	0.0021	9.8992E-35	0.000221505	150.9387755
rs9927848	A	C	-0.0122	0.002	6.4E-10	5.46154E-05	37.21
rs9951619	G	T	0.0156	0.002	1.3999E-15	8.92954E-05	60.84
rs998732	G	A	-0.0171	0.0022	1.9999E-14	8.86721E-05	60.41528926
rs9989141	T	C	0.0162	0.0017	3.6E-21	0.000133276	90.80968858
rs999889	A	G	-0.0108	0.0019	1.4E-08	4.7424E-05	32.31024931

Supplementary Table S6. Genetic instruments used in MR analysis of insuline treatment and preeclampsia

SNP	EA	OA	Beta	SE	p	R2	F-statistic
rs1002226	T	C	0.5154	-0.058	0.0106	3.96E-08	0.000136822
rs1046317	C	T	0.6107	0.0807	0.0109	1.04E-13	0.000250471
rs10466811	A	G	0.2372	-0.0704	0.0125	1.92E-08	0.000144956
rs10811660	A	G	0.1439	-0.1426	0.0151	4.24E-21	0.000407456
rs10882099	C	T	0.4766	-0.0896	0.0106	2.31E-17	0.000326464
rs112108223	A	G	0.02237	-0.3295	0.0371	6.17E-19	0.000360395
rs11257658	A	G	0.2648	0.0806	0.012	1.99E-11	0.000206153
rs112694524	A	G	0.03339	-0.1684	0.0299	1.76E-08	0.000144961
rs114322470	G	T	0.02355	-0.2359	0.0353	2.49E-11	0.000204075
rs11668971	C	T	0.5284	-0.0627	0.0106	2.89E-09	0.000159892
rs11709077	A	G	0.1707	-0.0984	0.0141	2.67E-12	0.00022255
rs2237897	T	C	0.08141	-0.172	0.0196	1.61E-18	0.000351856
rs2583921	C	A	0.0563	0.1315	0.023	1.06E-08	0.000149384
rs28624681	C	T	0.7078	0.0754	0.0117	1.05E-10	0.000189785
rs34337125	A	G	0.4142	-0.0603	0.0107	2.01E-08	0.000145136
rs3887925	T	C	0.463	0.0627	0.0106	3.30E-09	0.000159892
rs3957146	C	T	0.1197	0.4578	0.0164	1.40E-170	0.003548889
rs4273712	G	A	0.2689	0.0664	0.0118	2.08E-08	0.000144704
rs4481184	T	C	0.3081	0.082	0.0114	6.30E-13	0.000236422
rs45551238	T	C	0.0499	-0.2126	0.0247	7.85E-18	0.0003385
rs4858068	C	A	0.8459	-0.0839	0.0146	9.40E-09	0.000150912
rs6679677	A	C	0.1472	0.1288	0.0148	3.69E-18	0.000346043
rs6977081	T	G	0.3593	0.061	0.011	2.89E-08	0.000140535
rs74862545	T	C	0.01951	-0.261	0.0395	3.94E-11	0.000199514
rs76177300	A	G	0.05783	0.1409	0.0226	4.76E-10	0.000177624
rs76236532	A	G	0.04772	0.1625	0.0247	5.19E-11	0.000197788
rs76895963	G	T	0.03114	-0.4362	0.0331	1.05E-39	0.000793128
rs78868334	A	G	0.04924	0.1665	0.0243	6.73E-12	0.000214534
rs7903146	T	C	0.1996	0.2912	0.0134	1.33E-104	0.002153819
rs7910961	C	T	0.6783	0.0624	0.0113	3.50E-08	0.000139356
rs7998259	A	G	0.3894	-0.0665	0.0109	1.01E-09	0.000170094
rs8073177	C	T	0.7454	-0.0672	0.0121	2.90E-08	0.000140955
rs8100204	A	G	0.1599	0.1084	0.0145	8.44E-14	0.000255378

Supplementary Table S7. Genetic instruments used in MR analysis of metformin treatment and preeclampsia

SNP	EA	OA	Beta	SE	p	R2	F-statistic
rs10195252	C	T	0.405511	-0.08299	0.013835	1.99E-09	7.89E-05
rs10965246	C	T	0.176299	-0.17322	0.018063	8.81E-22	0.000202
rs11257655	T	C	0.208131	0.112948	0.016761	1.60E-11	9.95E-05
rs12889801	T	C	0.483099	-0.07827	0.013587	8.39E-09	7.27E-05
rs13094957	C	T	0.203842	-0.12401	0.016899	2.17E-13	0.000118
rs1359790	A	G	0.288055	-0.12047	0.015054	1.22E-15	0.00014
rs1483988	T	C	0.453187	-0.08652	0.013689	2.62E-10	8.75E-05
rs17036160	T	C	0.116603	-0.12904	0.021251	1.26E-09	8.08E-05
rs17250977	G	A	0.040121	0.190713	0.034977	4.96E-08	6.52E-05
rs1800961	T	C	0.031129	0.220696	0.039413	2.15E-08	6.87E-05
rs1801212	G	A	0.27999	0.113698	0.015167	6.55E-14	0.000123
rs2215383	T	C	0.450615	0.076542	0.013653	2.06E-08	6.89E-05
rs28746845	C	A	0.181708	0.09903	0.018166	5.00E-08	6.51E-05
rs34872471	C	T	0.290614	0.352165	0.015465	8.8194E-115	0.001135
rs459193	A	G	0.253179	0.093796	0.014943	3.45E-10	8.63E-05
rs4715207	T	C	0.179331	0.107482	0.01777	1.46E-09	8.02E-05
rs4932264	T	C	0.269847	-0.08915	0.015354	6.40E-09	7.39E-05
rs62106258	C	T	0.048384	-0.18424	0.03188	7.51E-09	7.32E-05
rs703965	T	C	0.453081	0.101184	0.013627	1.13E-13	0.000121
rs7177055	G	A	0.281595	0.089649	0.015106	2.95E-09	7.72E-05
rs7482891	A	G	0.378473	-0.09031	0.014038	1.25E-10	9.07E-05
rs7501939	T	C	0.397505	0.103221	0.013885	1.05E-13	0.000121
rs7615045	G	A	0.31476	0.12901	0.014663	1.39E-18	0.00017
rs76895963	G	T	0.014143	-0.47837	0.06138	6.52E-15	0.000133
rs77464186	C	A	0.156239	-0.11631	0.018712	5.10E-10	8.47E-05
rs780093	T	C	0.383721	0.086519	0.013931	5.29E-10	8.45E-05
rs849142	T	C	0.494467	-0.09788	0.013559	5.24E-13	0.000114
rs8756	C	A	0.484015	0.079568	0.013585	4.71E-09	7.52E-05
rs9410573	C	T	0.421132	-0.07732	0.013804	2.13E-08	6.88E-05
rs9957264	A	C	0.164876	-0.10474	0.018388	1.23E-08	7.11E-05
