# Supplementary figures and images for: Causal Associations Between Pre-Pregnancy Diabetes Mellitus and Pre-Eclampsia Risk: Insights from a Mendelian Randomization Study
Source: Healthcare (Basel). 2025 May 7;13(9):1085. doi: 10.3390/healthcare13091085 (PMC12072006; doi:10.3390/healthcare13091085)

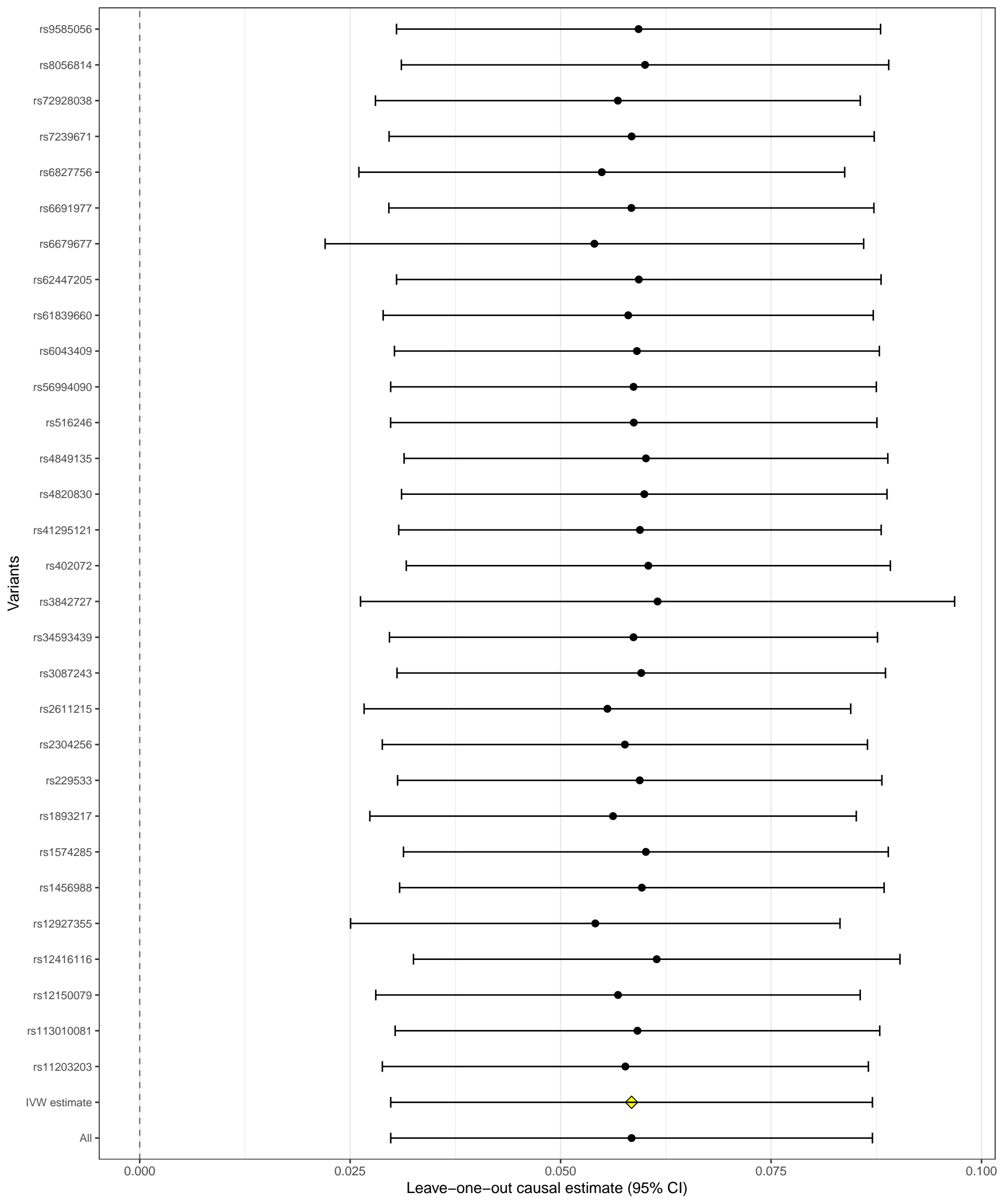

Supplement: Supplementary file 1 [file healthcare-13-01085-s001.zip › sup fig S1.pdf]

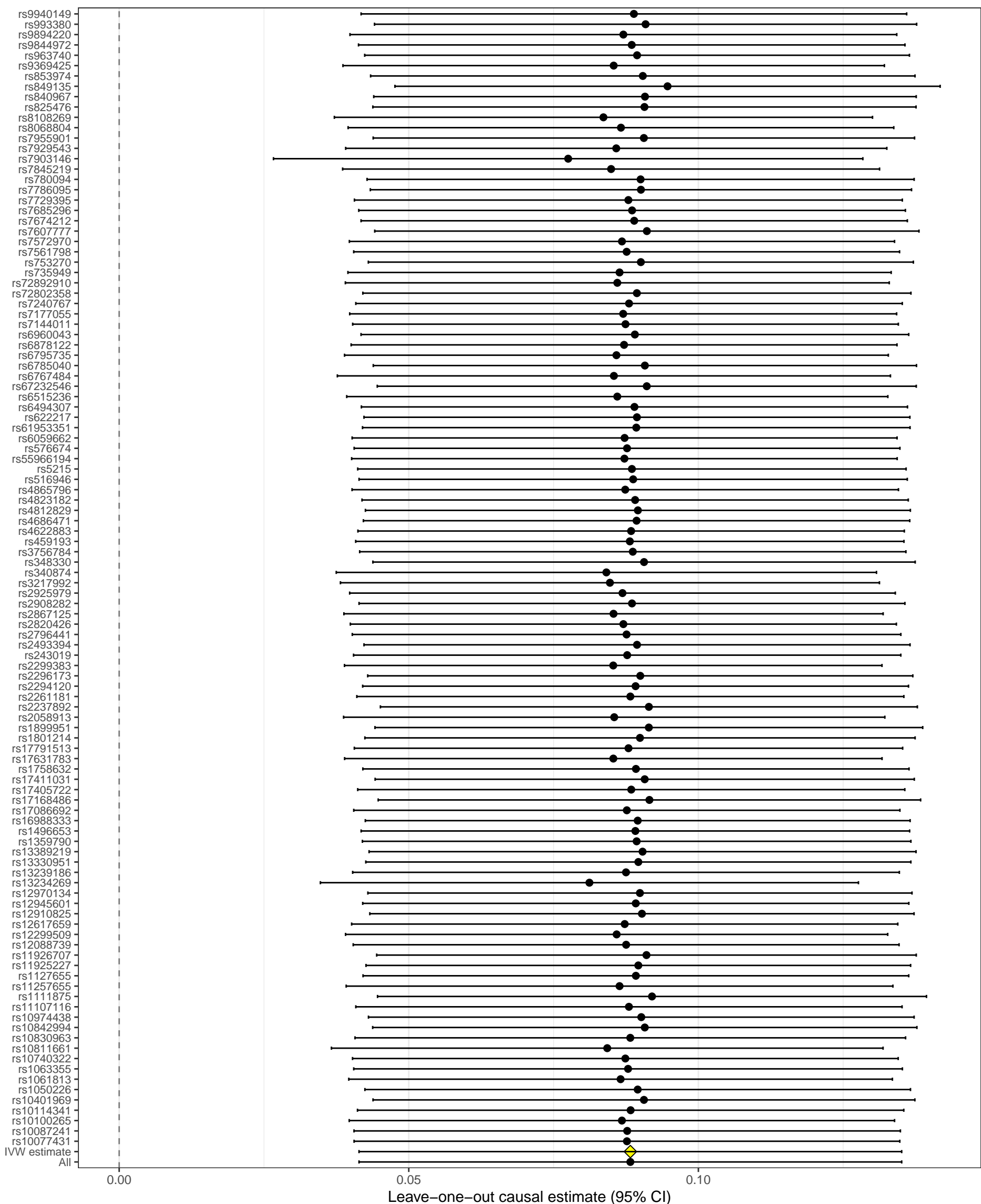

Supplement: Supplementary file 1 [file healthcare-13-01085-s001.zip › sup fig S2.pdf]

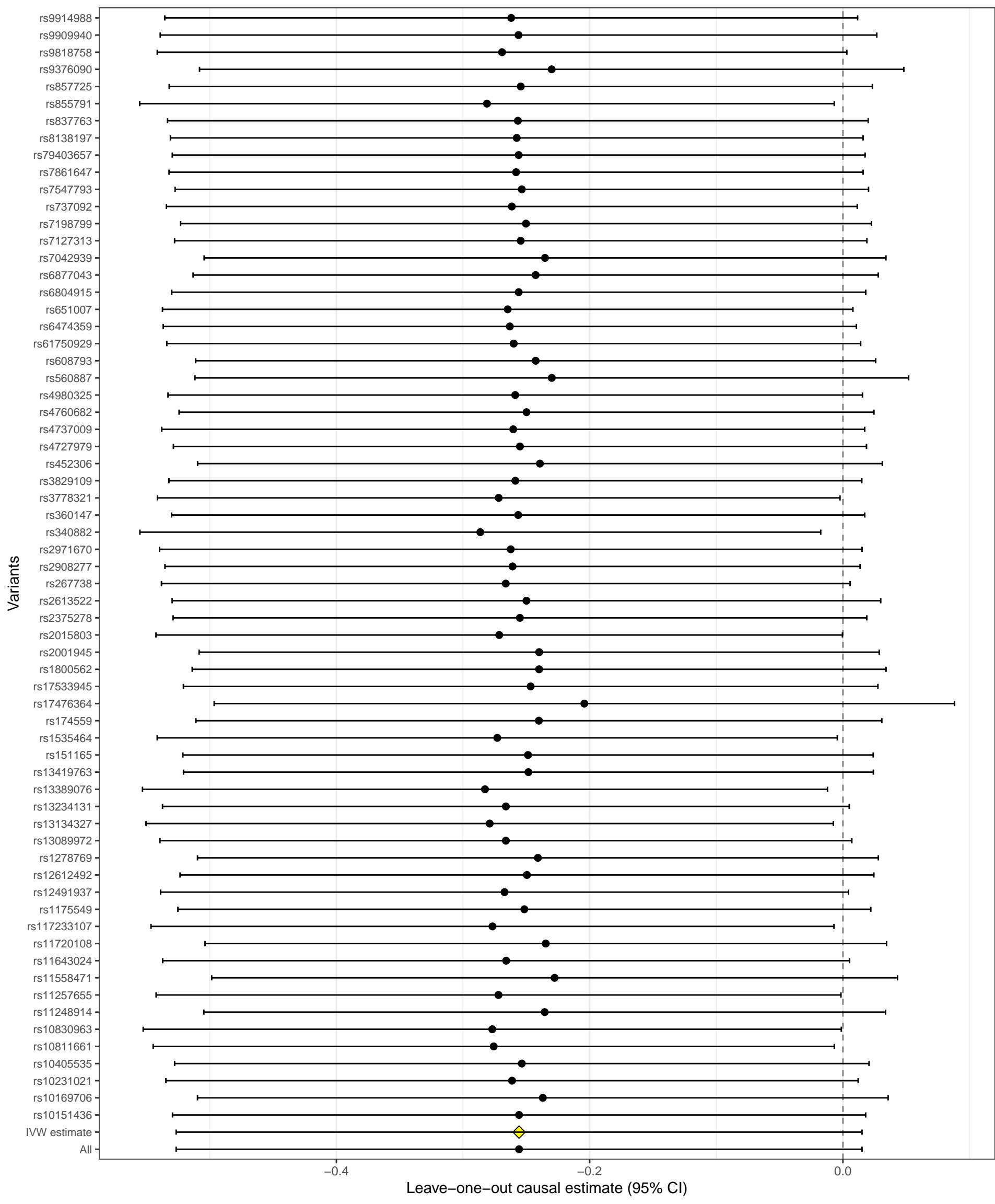

Supplement: Supplementary file 1 [file healthcare-13-01085-s001.zip › sup fig S3.pdf]

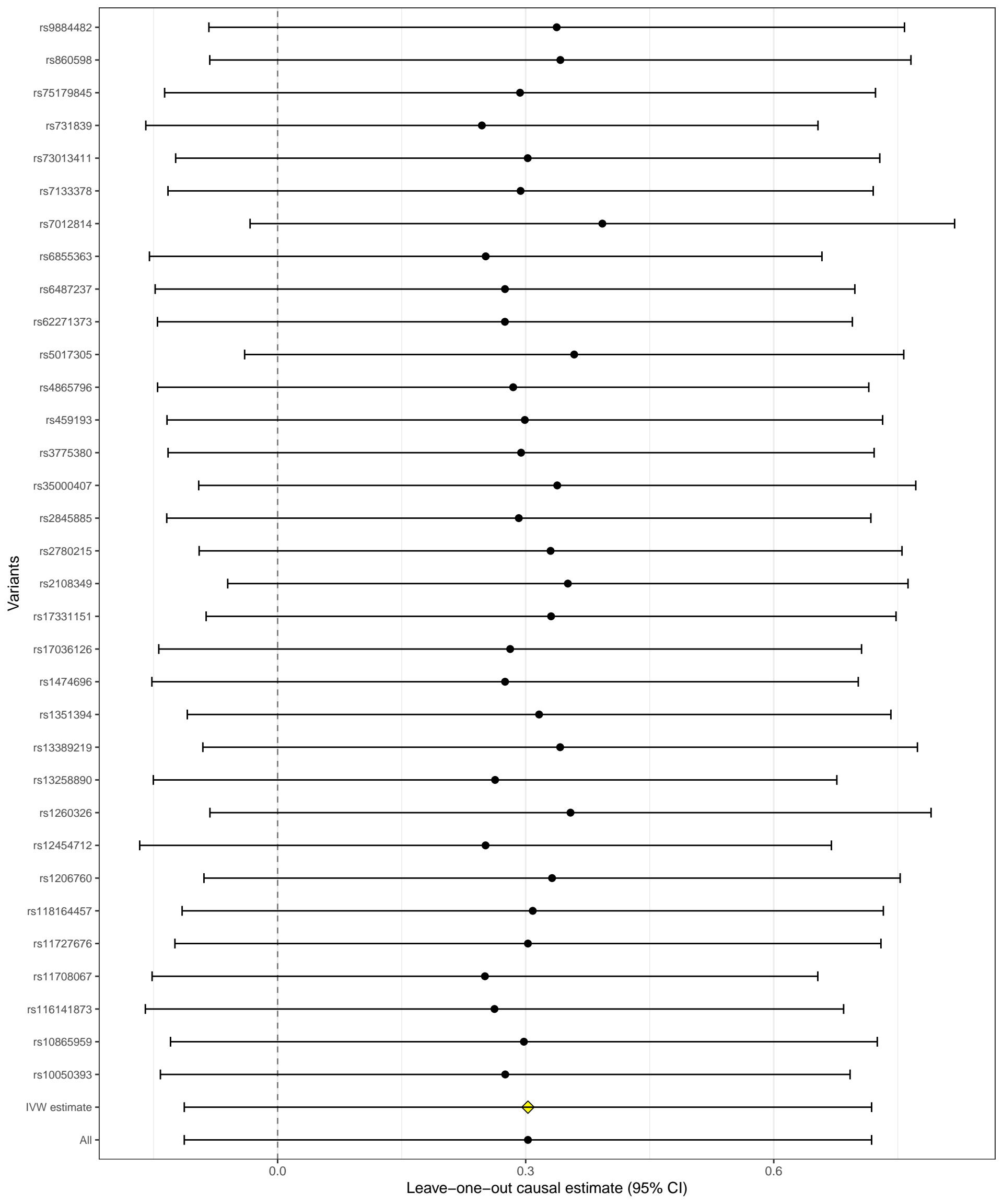

Supplement: Supplementary file 1 [file healthcare-13-01085-s001.zip › sup fig S4.pdf]

Variants

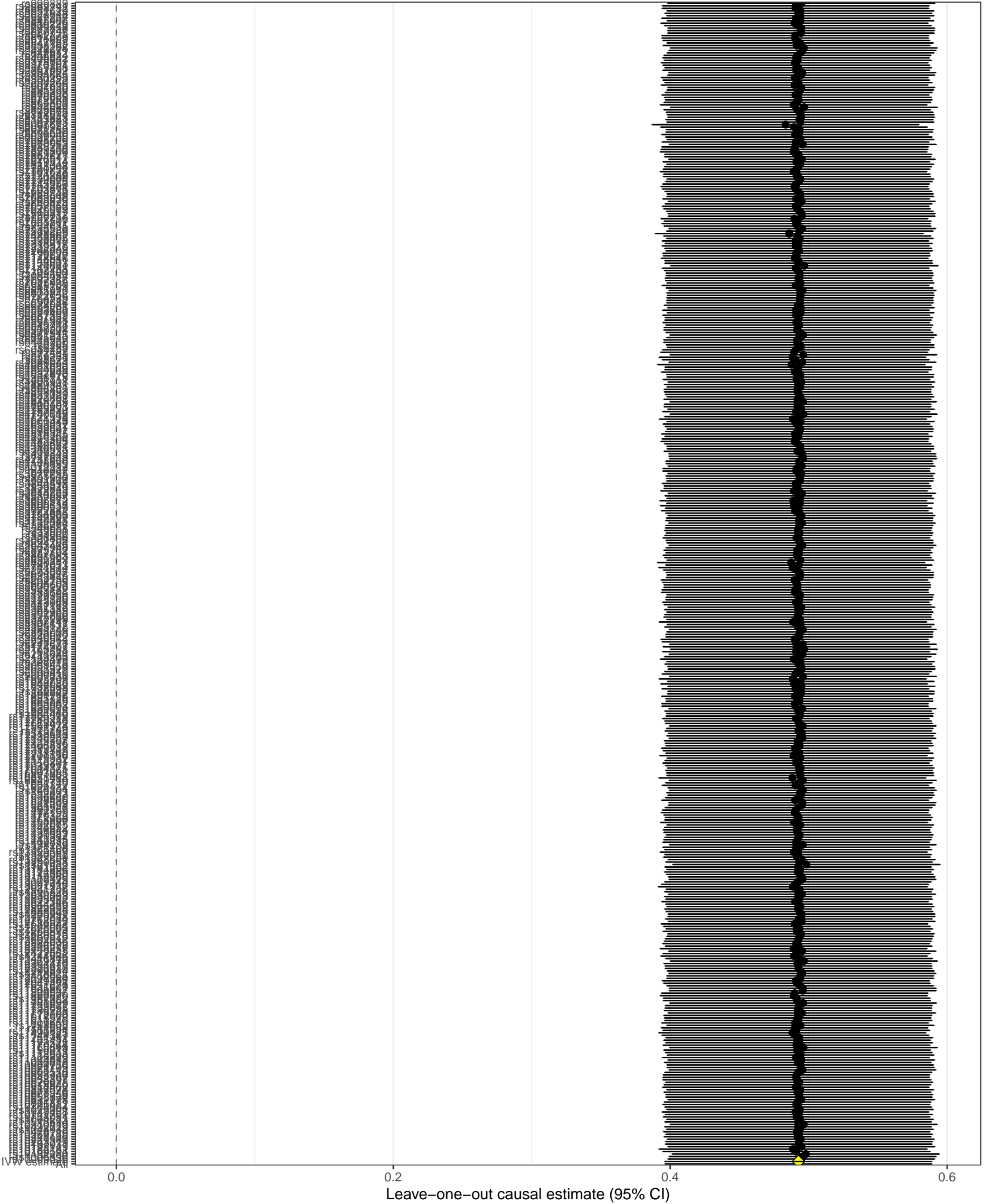

Supplement: Supplementary file 1 [file healthcare-13-01085-s001.zip › sup fig S5.pdf]

Antidiabetic Medications and Preeclampsia Risk

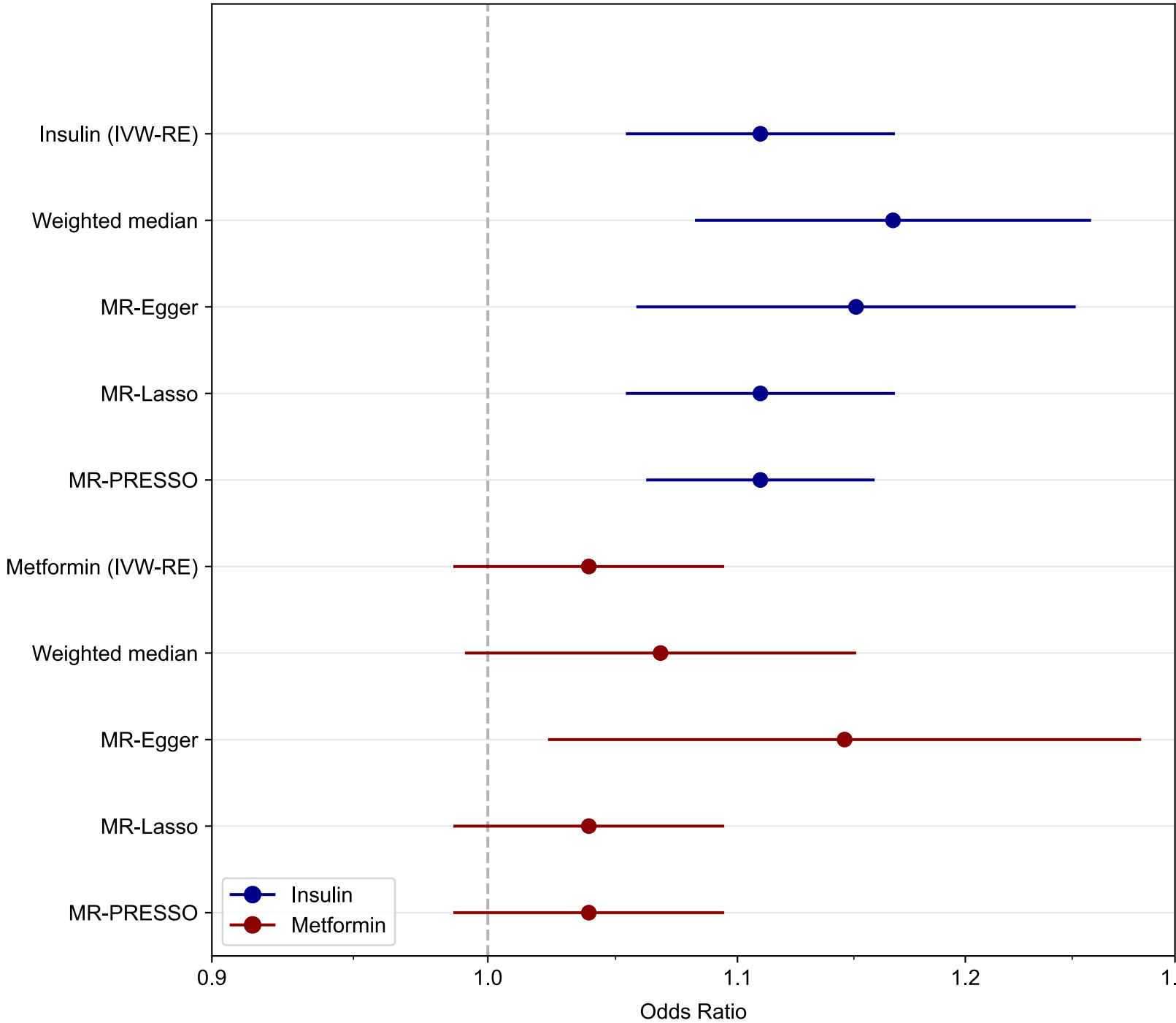

| Odds Ratio | 95% CI    | P-value |
|------------|-----------|---------|
| 1.11       | 1.05-1.17 | <0.001  |
| 1.17       | 1.08-1.26 | <0.001  |
| 1.15       | 1.06-1.25 | <0.001  |
| 1.11       | 1.05-1.17 | <0.001  |
| 1.11       | 1.06-1.16 | <0.001  |
| 1.04       | 0.99-1.09 | 0.140   |
| 1.07       | 0.99-1.15 | 0.081   |
| 1.15       | 1.02-1.28 | 0.018*  |
| 1.04       | 0.99-1.09 | 0.140   |
| 1.04       | 0.99-1.09 | 0.150   |

Supplement: Supplementary file 1 [file healthcare-13-01085-s001.zip › sup fig S6.pdf]
